# Allosteric Disulfide Bridges in Integrins: The Molecular Switches of Redox Regulation of Integrin-Mediated Cell Functions

**DOI:** 10.3390/antiox14081005

**Published:** 2025-08-16

**Authors:** Johannes A. Eble

**Affiliations:** Institute of Physiological Chemistry and Pathobiochemistry, University of Münster, Waldeyerstr. 15, 48149 Münster, Germany; johannes.eble@uni-muenster.de; Tel.: +49-251-8355591

**Keywords:** allosteric disulfide bonds, integrin, redox regulation, conformational signaling

## Abstract

Almost every cell of a multicellular organism is in contact with the extracellular matrix (ECM), which provides the shape and mechanic stability of tissue, organs and the entire body. At the molecular level, cells contact the ECM via integrins. Integrins are transmembrane cell adhesion molecules that connect the ECM to the cytoskeleton, which they bind with their extracellular and intracellular domains. Cysteine residues are abundant in both integrin subunits α and β. If pairwise oxidized into disulfide bridges, they stabilize the folding and molecular structure of the integrin. However, despite the oxidative environment of the extracellular space, not all pairs of cysteines in the extracellular integrin domains are permanently engaged in disulfide bridges. Rather, the reversible and temporary linkage of cystine bridges of these cysteine pairs by oxidation or their reductive cleavage can cause major conformational changes within the integrin, thereby changing ligand binding affinity and altering cellular functions such as adhesion and migration. During recent years, several oxidoreductases and thiol isomerases have been characterized which target such allosteric disulfide bridges. This outlines much better, albeit not comprehensively, the role that such thiol switches play in the redox regulation of integrins. The platelet integrin αIIbβ3 is the best examined example so far. Mostly referring to this integrin, this review will provide insights into the thiol switch-based redox regulation of integrins and the known effects of their allosteric disulfide bridges on conformational changes and cell functions, as well as on the machinery of redox-modifying enzymes that contribute to the redox regulation of cell contacts with the ECM.

## 1. Introduction

Cysteine residues are the most conserved amino acids in proteins, even more conserved than tryptophan [1]. This is presumably due to their structure-stabilizing effect on proteins. Two cysteines that are spatially close-by can be oxidized to form a disulfide bond. This covalent bond is among the strongest bonds that can stabilize tertiary and sometimes quaternary structures, if formed as intra- and intercatenary links, respectively. This stabilizing effect on protein structure is important and worthwhile enough to be evolutionarily preserved [1], especially if reductive cleavage of a cysteine bridge would make a protein lose its structure and concomitantly its function. However, not every pair of vicinal cysteines serves such a structurally indispensable role. In this review, light is shed on cell adhesion-mediating molecules, especially integrins, in which, along with several structure-stabilizing cysteine bridges, cysteine pairs also exist that, due to reversible formations of disulfide bonds and their cleavage, act as thiol switches and regulate protein function.

A redox pair, such as two cysteines and cystine, only undergoes a redox reaction if a convenient oxidant or reductant with an appropriate redox potential thermodynamically allows an electron transfer and is kinetically able to react in reasonable time [2]. Therefore, the oxidative formation of a disulfide bridge or its reductive cleavage depends on its environment, including redox-active compounds and thiol group-modifying enzymes [3]. Moreover, in order to allow redox regulation, this environment has to be altered by the cell. The cytoplasm of a cell remains largely at a low, hence, reducing redox potential due to the GSH/GSSG and cysteine/cysteine buffer with high molar fractions of the reduced species [4,5]. In contrast, certain compartments within the cells, such as mitochondria, endoplasmic reticulum (ER), Golgi organelle and transfer vesicles, as well as the extracellular space, have higher redox potentials [6,7,8]. Moreover, in addition to the access to oxidizing molecular oxygen, certain cells can produce highly oxidative species such as reactive oxygen species (ROS) and reactive nitrogen species (RNS) upon certain stimuli which increase redox potential locally and temporally [9,10,11]. As an example, vascular smooth muscle cells transiently produce pulses of ROS locally and thus form redox hot spots at sites of adhesion [12]. NADPH oxidase (Nox) isoforms, preferentially Nox4, are recruited to these local cell protrusions, where integrins also gather to form adhesive contacts with the ECM. Conversely, these oxidants produced by the cells cause the formation of disulfide bridges within the integrins, especially at their allosteric thiol switches, which will be highlighted in this review.

## 2. Not All Cysteine Pairs in Proteins Are Equal

Disulfide bridges within proteins and their formation have been considered as a path-determining step in protein folding and as a stabilizing element for the tertiary and quaternary structures of proteins, especially those in the extracellular space, where the redox potential generally is higher than in the cytoplasm due to the higher oxygen tension and low concentration of redox buffers such as glutathione [3]. Thus, they have been regarded as static elements of protein structure, referred to as structural cysteines. However, labeling of extracellular proteins with thiol-selective mass spectrometric probes, which differ in their isotope composition, before and after reductive cleavage of disulfide bridges (differential cysteine alkylation), revealed that a remarkable fraction of vicinal cysteine residues, albeit in a distance of the two thiol groups close enough to form a disulfide bridge (0.28 nm), were not oxidized to a disulfide bridge. Moreover, the fraction of disulfide-linked and reduced cysteine residues varied for each cysteine pair at different sites of these proteins [13]. Interestingly, a comprehensive study of the protein structure database revealed that many proteins contain such pairs of cysteines, which form labile and mechanically stressed disulfide bridges and tend to be reversible and temporal [14]. In addition, these disulfide bridges can induce conformational changes within the protein during their formation and cleavage and have therefore been termed allosteric disulfide bonds [15,16]. Such cysteine pairs are mostly located at or close to the protein surface and thus are accessible to redox-modifying enzymes.

Many extracellular proteins bear such cysteine pairs, some of which are not necessarily oxidized into a linking disulfide bridge, or some of which can be temporarily crosslinked by oxidation and cleaved by reduction [15,17,18,19]. Such allosteric disulfide bridges can be found in several of the coagulation factors and other blood proteins [20], such as fibrinogen, α2 macroglobulin [21], von Willebrand factor (vWF), Histidine-rich glycoprotein (HRG) [17], and in several extracellular domains of hemostasis-relevant membrane proteins, such as tissue factor [22,23], vWF receptor GPIbα [24] and integrins [25].

Along with the finding of allosteric cysteine pairs in extracellular proteins, several redox-modifying enzymes, originally thought to be localized within the cells and the lumen of vesicular organelles, such as ER and Golgi, have been detected in the extra- and pericellular space, among them protein disulfide isomerase (PDI), several thiol isomerases, thioredoxins, peroxiredoxin-4 (Prx4) and quiescin sulfhydryl oxidase-1 (QSOX1) [3,26,27]. They serve functions in the redox regulation of proteins, both under physiological and pathological conditions, such in normal thrombus formation [28] and cancer-induced thrombosis [29].

## 3. Adhesion of Platelets and Cells Are Mediated by Members of the Cysteine-Rich Integrin Receptor Family

One of the best examples of how cellular adhesion is regulated by the redox properties of the environment is platelet aggregation at injured blood vessels, the initial step of hemostasis that is followed by coagulation and thrombus formation [29,30,31,32]. Platelets are anucleate cell fragments pinched off from megakaryocytes in the bone marrow and float in the blood stream during their life span of 8–10 days [33]. After stimulation by soluble procoagulants, such as purine nucleotides or proteases, they become activated and change their morphology from a discoid to protrusion-rich stellate shape. Moreover, they rearrange their cytoskeleton, degranulate their vesicles, and activate the fibrin-binding platelet integrin αIIbβ3 [33]. Another stimulus for platelet activation is the contact with accessible extracellular matrix molecules, such as collagens from the subendothelial layers of blood vessels [34]. Six different integrin receptors are expressed on the platelet surface [35]. With its 80,000 copies per platelet, integrin αIIbβ3 outnumbers any other integrin receptor, hence it is also called ‘the platelet integrin’. After activation, the integrin αIIbβ3 binds fibrin bundles that have been formed as a result of blood coagulation, resulting in a firm fibrin–platelet clot, as well as in stabilization and contraction of the thrombus [36]. Its binding activity is regulated not only by ligand occupancy, but also by intracellular adaptor molecules [37] and extracellular redox-modifying agents and enzymes [28,30].

Along with 23 other members, the integrin αIIbβ3 belongs to the integrin family, all of which consist of two non-covalently associated and glycoconjugate-bearing subunits, α and β [38,39]. Eighteen α and eight β subunits, most of them specified by a number (α1 through α11 and β1 through β8) or acronyms (αV for the vitronectin-binding integrins, and αIIb for the platelet glycoprotein IIb), heterodimerize within the endoplasmic reticulum during their biosynthesis and form receptors on the cell surface [40,41]. The integrins containing the β1, β3, and β4 subunit recognize proteins of the extracellular matrix (ECM) [42,43], thereby anchoring cells to the extracellular scaffold and transmitting mechanical forces and environmental cues between the cells and the ECM scaffold [44]. According to their ECM ligands, subgroups are defined as collagen-binding (α1β1, α2β1, α10β1, and α11β1) and laminin-binding integrins (α3β1, α6β1, α6β4, and α7β1). They recognize their ligands by an array of amino acids presented in a characteristic three-dimensional structure, such as the triple helix of collagens and the globular domains of laminins [45,46]. In contrast, RGD-dependent integrins bind their ligands via the characteristic tripeptide motif, Asp-Gly-Arg (RGD in the one-letter-code), such as the fibronectin receptor α5β1, as well as the integrins α8β1, α9β1, αV-subunit containing integrins, and the platelet integrin αIIbβ3 [40,41,45]. The integrins containing the β2 integrin subunit, αLβ2, αMβ2, αXβ2, and αDβ2, are expressed on leukocytes and are often grouped with other immunologically relevant integrins (α4β1, α4β7, and αEβ4). They mediate cohesive cell–cell contacts with endothelial cells during lymphocyte patrolling and homing or with E-cadherin of epithelial cells during immune surveillance. Some of them are also involved in pathogen defense as they significantly contribute to the phagocytosis of complement-opsonized pathogens [47,48].

Integrin-mediated signaling in platelets is a well-studied example as of how cells sense and regulate adhesive interactions with the ECM via integrins [25,33,49]. On quiescent thrombocytes, the platelet integrin αIIbβ3 is surface-exposed but does not bind to its ligand, fibrinogen, which is abundant in the blood plasma. Upon damage to the vessel wall, ECM proteins, such as collagen fibrils, become accessible to and are recognized by thrombocytes via different receptors, among them the collagen-binding integrin α2β1 [50,51]. This triggers an outside-in signaling cascade within the platelets, which results in release of additional platelet-activating substances, cytoskeletal rearrangement, and inside-out activation of the platelet integrin αIIbβ3 via association of cytoplasmic integrin-associating proteins, such as kindlins [33,37,49,52]. Only activated αIIbβ3 binds to fibrinogen and its coagulation-converted product, fibrin. Via the integrin αIIbβ3-fibrin axis, thrombocytes adhere to the fibrin network and keep the fibrin network under stabilizing tension, thus closing the bleeding wound [53].

Upon ligand binding, integrins coral into clusters and recruit additional adaptor and signaling proteins to their cytoplasmic domains [54,55,56]. Thus, in an adhesion-dependent manner, new adhesive supramolecular complexes, called adhesomes, are formed, among which focal contacts and focal adhesion (plaques) at the cell periphery during cell spreading and migration can be distinguished from fibrillary adhesions underneath the cell soma [57,58]. They all contain distinct signaling and adaptor proteins and cytoskeleton-linking molecules that enable the integrins in the adhesome to transmit mechanical forces and convey signals between cells and their environment [44,56]. Noteworthy is that not only the integrins, but also integrin-associated adaptor molecules within focal adhesions [59,60] and several ECM proteins are subject to redox modifications [14,47,48,61].

## 4. Structure; Domains and Disulfide Pattern of Integrins

Integrins are characterized by a high number of cysteine residues, which are distributed throughout the extracellular domains of both subunits. While the integrin β subunits bear more than 50 cysteine residues, the α subunit still has a high number of about 20 cysteine residues. Not all of them engage in disulfide bridges, and the fraction of disulfide bond formation varies for each cysteine pair [25].

**Figure 1:**
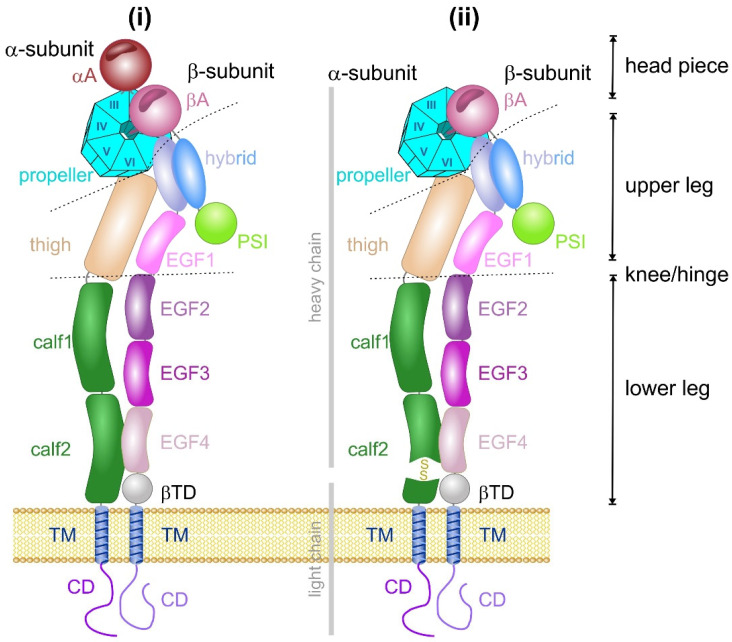
Domain structure of integrins that do (i) or do not (ii) contain an A-domain in their α subunit. 24 integrins are known so far, all of which consist of two subunits, α and β. Half of the 18 different α subunits carry an A-domain inserted between blades II and III within the propeller domain. The domain names of both integrin subunits are labeled. The domains comprise several functional units: the head piece and the upper and lower legs. The head piece binds the ECM ligand and consists of the propeller domain and, if present, the α subunit A-domain (αA), as well as the A-domain of the β-subunit (βA), which is anchored to the propeller domain via the side chain of a basic amino acid residue. The domains of the β subunit are plexin-semaphorin-integrin (PSI), hybrid, four epidermal growth factor-homology (EGF), βtail-domain (βTD) and, like in the α subunit, a single membrane-spanning transmembrane (TM) and cytoplasmic domain (CD). The calf-2 domain of the integrin α subunits, which lack an A-domain, is proteolytically processed and cleaved into a heavy and light chain that are held together via an interchain disulfide bridge.

Both subunits are type I membrane proteins, each with large N-terminal ectodomains, which are anchored by a single-pass transmembrane domain. The α and β subunits end with a short C-terminal cytoplasmic domain of 20–50 and 15–65 residues, respectively (Figure 1). Only the cytoplasmic tail of the β4 chain extends to more than 1000 amino acids and contains four fibronectin type II domains arranged in two tandem pairs. The cytoplasmic domains harbor binding sites for various adaptor and signaling molecules that are relevant for integrin-mediated signal transduction and mechanical force transmission [56,58,62]. Reversible phosphorylation of tyrosine residues within the integrin β cytoplasmic tail regulates interactions with several of these adaptor and signaling molecules [63,64,65]. In non-activated integrins, the transmembrane domains of both subunits heterodimerize via several hydrophobic interactions and an interchain salt bridge between membrane-proximal cytoplasmic residues [66,67], whereas integrin activation causes the two different transmembrane domains to separate from each other [68,69].

The molecular structure of the cysteine residue-bearing ectodomains of the integrin αVβ3 was the first to be resolved by crystallographic X-ray diffraction analysis in 2001 [70], followed by the ectodomains of other αV-integrins, α5β1 and β2 integrins, and eventually of the entire αVβ3 and αIIbβ3 heterodimer [71,72,73,74]. Both integrin subunits consist of several domains. The N-terminal domain of the mature integrin α subunit is the propeller domain made of seven blades, each with four β strands, and a central pore [70]. Between the second and third blade, an additional A-domain is inserted in about half of the integrin α subunits, especially in the collagen-binding and β2 integrin subfamilies (Figure 1). Since a magnesium ion serves as complex-forming bridge for the binding of the ECM ligand, the ligand binding crevice located on top of the A-domain is referred to as a metal ion-dependent binding site (MIDAS) [75,76,77]. In addition to the Mg^2+^ ion, two Ca^2+^ ions are complexed in close vicinity to the Mg^2+^ ion and shape the so-called adjacent metal ion binding site (ADMIDAS) and synergistic metal binding site (SyMBS) [78]. In those integrin α subunits that lack an A domain the propeller binds directly to the ECM ligand. The last four blades of the propeller domain also complex Ca^2+^ ions. However, these metal ion binding sites likely serve structural purposes as they are located on the bottom face of the propeller domain opposite the ligand binding site. The propeller domain and, if present, the A-domain of the α subunit, together with the A-domain of the β subunit, shape the integrin headpiece. Therein, the propeller domain forms the rather inflexible structural foundation. C-terminally adjacent to the head domain, the leg/stalk of the integrin α subunit is formed by a β strand-rich thigh domain and two calf-domains, calf 1 and 2. In integrin α subunits lacking an αA-domain, the calf 2 domain is usually cleaved proteolytically into the N-terminal heavy and C-terminal light chains, which remain covalently cross-linked via a disulfide bridge (Figure 1). As an exception, integrin α4 is cleaved within the thigh domain.

In the ectodomain of the integrin β subunit, the peptide chain starts with the N-terminal plexin-semaphorin-integrin (PSI) domain, passes through the hybrid domain to the βA-domain, and passes a second time through the hybrid domain connecting the βA-domain with the EGF1-domain (Figure 1). The βA-domain is inserted between the first and second peptide chain passage through the hybrid domain. The A-domains of both integrin subunits show homologous folding and complex a divalent cation on their top. Interestingly, a conserved basic amino acid side chain, R261, of the βA-domain points into the central pore of the propeller domain of the integrin α subunit [70]. In addition, hydrophobic side chains, especially those of phenylalanine residues, also interact between the propeller and A domains of both subunits that thus jointly form the integrin head domain [79].

The stalk of the integrin β subunit is shaped by 4 EGF domains, each of which contains three or four disulfide bridges, and by a membrane-proximal β-tail domain (βTD), which, despite its distance from the ligand-binding head domain, also influences ligand-binding activity by interacting with the βA-domain in one of the integrin conformations [80,81]. Moreover, the conformation changes that occur during integrin activation and ligand binding alter the distance and orientation of the integrin stalk/leg domains [82].

The location and pair-wise distances of cysteine residues and the resulting disulfide pattern within both integrin subunits had been a thrilling conundrum. Early protein chemical and mass spectrometric analysis mapped most of the 28 and 9 potential disulfide bridges within the integrin β and α subunits, respectively, of the platelet integrin [83,84,85]. However, data from the crystallographic protein structure determination showed several discrepancies regarding the location of some disulfide bonds [74]. These discrepancies might be due to conformational differences, as crystallography has only revealed the structure of one out of various integrin conformations.

**Figure 2:**
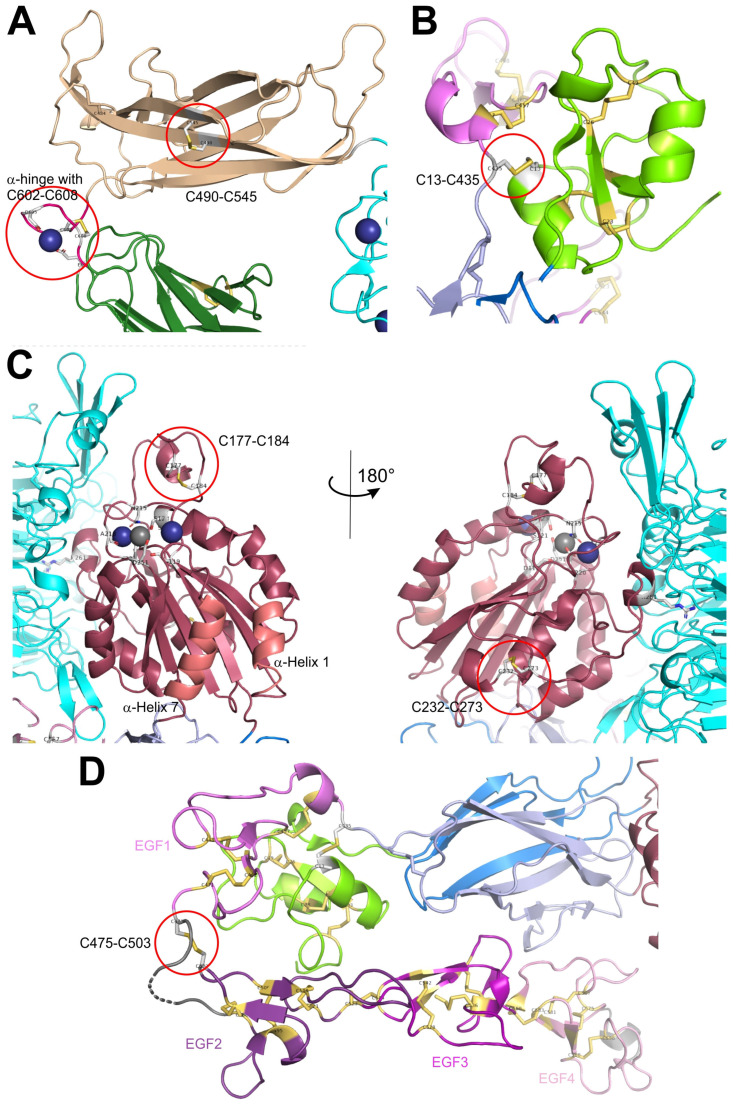
Location of allosteric disulfide bonds within the integrin domains. (**A**) The two thiol switches within the thigh (wheat) and hinge (red) domains of the integrin α subunit. Although the two cysteines, C490 and C545, in the thigh domain are mostly reduced [25] and allow flexibility, both residues are located in two neighboring strands within one β sheet and thus appear to be in a structurally fixed position without much conformational flexibility. Nevertheless, this cysteine pair is highly accessible on the surface of the entire protein. The thiol switch, C602–C608, located in the hinge domain of the α subunit that flanks the upper leg and the calf 1-domain of the lower leg (dark green) is crucial because it determines the conformational change of the integrin ectodomains between the bent and extended conformation. The hinge sequence embraces residues that together with residues of the calf 1 domain complex a Ca^2+^ ion. (**B**) PSI domain of the integrin β domain (green) with its long distance interdomain disulfide bridge C13–C435 to the EGF1 domain (magenta). Although the cysteines C13 and C16 of the PSI domain show a sequence homology to the CXXC motif of thiol isomerases, both cysteines are not engaged with themselves but with other cysteines within the EGF1 and the PSI domain. (**C**) Side views of the βA domain (raspberry) with its two redox-relevant cysteine pairs, C177–C184 and C232–C273. The Mg^2+^ ion (grey sphere) and two Ca^2+^ ions (dark blue spheres) mark the top of the A-domain and are complexed by residues in the loops connecting the β strands of the central β-sheet with each other or with the α-helices that flank the central β-sheet on either side. The βA-domain is firmly anchored to the integrin α subunit via the R261 residue, that penetrates deeply into the central pore of the propeller domain (cyan). Visible from one side (i) are the first and last α-helices, 1 and 7, that are connected closest to the peptide chain that enters from (navy blue) and leaves into (lilac blue) the respective peptide chains of the hybrid domain. The redox-regulated thiol switch, C177–C184 (encircled in red), is located in the second loop between β-strands A and B, on top of the A-domain in close vicinity to the divalent cation-binding site. Visible after a 180° rotation around the long axis of the A domain (ii), the cysteine bridge, C232–C273 (encircled in red), is located in a loop that is directly connected to the R261 residue, which mediates the firm interaction of propeller domain and βA domain within the integrin head piece. (**D**) The disulfide-rich EGF-domains, EGF1–EGF4, of the integrin β subunit (in different hues of magenta). While EGF1 is part of the upper leg, together with the hybrid domain (navy blue and lilac blue) in the upper part of the image, the domains EGF2, EGF3 and EGF4 form the lower leg, shown in the bottom part of the panel. The hinge region of the β subunit, which connects EGF1 with EGF2, contains another redox-regulated disulfide bridge, C475–C503 (encircled in red), and is close to the PSI domain with its long-range disulfide bridge to the EGF1 domain (see also (**B**)). The molecular structure was rendered with Pymol Molecular Graphics System software, version 2.3.1, using the protein data file 3FCS.pdb of RCSB Protein Data BANK.

In the primary sequence of both ectodomains of integrin αIIbβ3, cysteines are comparatively abundant with 20 to 35 and between 50 and 60 residues in the α and β subunit, respectively [85]. Within the α-chain, cysteines are localized in the first three blades of the propeller domain (C56–C65, C107–C130, C146–C167, with numbering referring to the αIIB subunit); however, in non-homologous strands or linker sequences. They are localized within each blade and likely serve structural functions. The thigh domain and calf 1 domain contain four and two cysteine residues, respectively, engaged in two (C475–C484, C490–C545) and one (C674–C687) disulfide bridges, respectively (Figure 2A). These two domains flank the α subunit hinge region, which contains a pair of cysteines (C602–C608) and complexes one divalent metal cation. The disulfide pattern between the four cysteines (C826, C880, C885, and C890) within the calf 2 domain differ between the crystallographic and protein–chemical analyses [74,83]. Being oxidizable by hydrogen peroxide, these cysteines are likely not fully engaged in disulfide bridges [12,13,25]. Interestingly, the C-terminal cysteine within the heavy chain (C826) is covalently crosslinked with one of the other three cysteines of the calf 2 domain, located within the light chain. This disulfide bridge holds the heavy and light chains of the integrin α subunit together and hence is of structural relevance (Figure 1).

Within the β-subunit, eight cysteines are located within the N-terminal PSI-domains [86]. Six of them form intradomain disulfide links (C5–C23, C16–C38, and C26–C49, with numbering referring to the β3 subunit), whereas the second cysteine, C13, forms a long-range disulfide bond with a cysteine, C435, situated more than 400 residues C-terminally within the first EGF-domain [70,74,86,87] (Figure 2B). Both the βA and hybrid domains contain four cysteines each, which, as cysteine bonds (C177–C184 and C232–C273 in the βA-domain), stabilize the globular and rod-like structures of these domains but may also serve regulatory functions (Figure 2C). Two disulfide bridges, C374–C386 and C433–C443 of the hybrid domain, crosslink the peptide chain on its second passage through the hybrid domain. Within the four EGF-domains, especially the last two of them, the cysteines share a common folding pattern in the order 1–5, 2–4, 3–6, and 7–8 [88,89]. Since the EGF1 domain is connected to the PSI domain via the long-range disulfide bond C13–C435 with its first cysteine, C435, it, exceptionally, has only three intradomain cystine bridges. Its last cysteine, C472, forms an interdomain disulfide bridge, C473–C503, to the first cysteine, C503, of the EGF2 domain, thereby spanning the domain-connecting loop between EGF1 and EGF2 (Figure 2D), which is the very flexible hinge region of the integrin β subunit [70,74,89]. This is in conspicuous contrast to the common disulfide pattern of other EGF-domains [90,91]. The ten cysteines of the βTD form engage in well-defined intradomain disulfide bridges.

**Figure 3:**
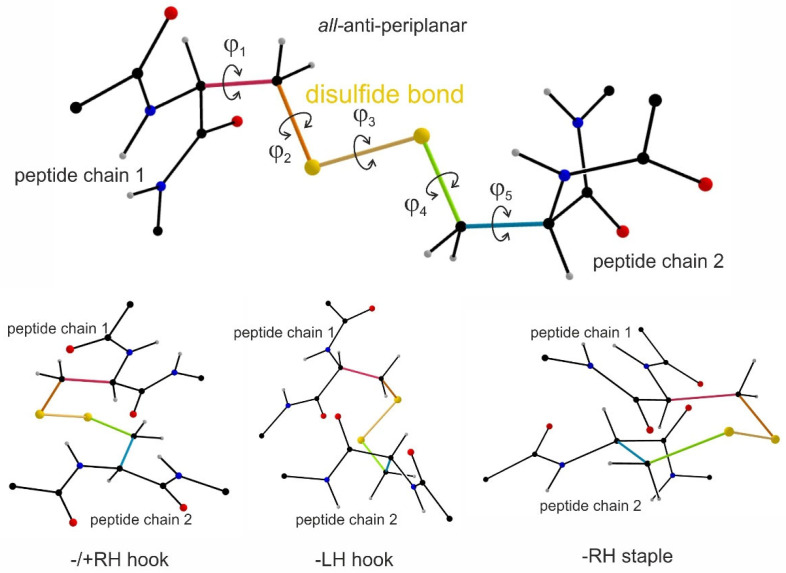
Stereochemistry of conformers of disulfide bond-linked peptide chains and the most prominent conformations of allosteric disulfide bridges. Two cysteine-containing peptide chains can form a disulfide bond, marked in yellow. Like the four freely rotatable single bonds between the αC- and βC-atoms and between the βC-atoms and the sulfur atoms (yellow), the disulfide bond (highlighted in yellow) between the sulfur atoms can also rotate around its single bond. These five bonds are indicated in rainbow colors and the dihedral angles, which these bonds may take, are labelled by φ_1_ through φ_5_. For clarity, the conformation, in which all dihedral angles lead to an anti-periplanar conformation is shown in the upper panel, which is likely to have the lowest conformational energy. The second lowest conformational energy level is taken by conformers in syn gauche conformations, which are reached from the anti-periplanar conformation by turning the dihedral angles, φ_1_, φ_2_, φ_3_, φ_4_, and φ_5_, around 120°, 120°, 97°, 120°, 120°, either clockwise (+) or counterclockwise (-), resulting in two possible conformers for dihedral angles. As compared to the distribution of more than 13,000 disulfide bond conformations in protein data banks [15], allosteric disulfide bonds show a significant over-representation of three typical conformers, -/+RH hook (+,+,+,-,-), -LH hook (-,+,-,-,-), and -RH staple (-,-,+,-,-), with the rotational orientation of the five dihedral angles given in parentheses. The dihedral strain energies represent the mechanical tension of the disulfide bond. They range from 12.8–14.7 kJ/mol, 15.1–17.1 kJ/mol, to 17.3–18.8 kJ/mol for these three conformers, with the latter two being above the average of all disulfide conformations of 14.5–15.0 kJ/mol [16]. The images were rendered with GeoGebra Classics software, version 6.0.892.0.

Previous studies assumed that a pair of cysteine residues must be engaged within a disulfide bridge in an all-or-none fashion. However, more recent differential alkylation studies, in which thiol groups in integrins were detected with two isotopically differently labeled 2-iodo-N-phenylacetamides (IPA) before and after reductive cleavage of disulfide bridges [13,25,92], revealed that the pairs of cysteines in integrins are not completely oxidized into cysteine bridges and, in fact, that each pair of cysteines has a distinct ratio of disulfide bridge formation [25], explaining some of the differences in disulfide patterns within integrins of previous studies. Additional studies revealed that certain cysteine pairs are more prone to redox-mediated disulfide engagement when exposed on the protein surface, indicating their accessibility to redox-modifying agents or enzymes [25]. The analysis of thousands of cysteine bonds in protein structure data bases revealed that in proteins, cysteine bonds can take 20 different conformations depending on the 5 dihedral angles of the five rotatable single bonds between the αC-atoms of two cysteine residues engaged in such a cysteine bond [14,15,16] (Figure 3). Three conformers are the main representatives within the group of allosteric disulfide bonds, which are called/+ RH hook, -LH hook, and -RH staple [15] (Figure 3 bottom panel). Together, they make about 80% of the allosteric disulfide bonds, while their distribution frequency within several thousands of disulfide bonds, irrespective of their structural or regulatory role, only run to 7.20%, 7.48%, and 6.44%, respectively, in total about 20% [16]. The -RH staple conformation has an exceptionally high dihedral strain energy, a measure for the mechanical tension that they withstand to hold the peptide chains in the tertiary structure of the protein. High dihedral strain energy values also indicate instability of the disulfide bond and its tendency to be cleaved. Thus, a group of redox-labile cysteines could be identified also in the integrin αIIbβ3, which are likely to be reversible oxidized to disulfide bridges and again reduced into free thiol groups, depending on external factors [15,25]. These novel insights have opened a new field of research: whether and how the formation and cleavage of these particular allosteric disulfide bridges is regulated in a redox-dependent manner, as well as which consequences the oxidation and reduction of cysteines and disulfide bridges, respectively, may have for regulation of integrin activation.

## 5. The Machinery That Modifies Integrins on the Cell Surface and Redox-Regulates Platelet Adhesion and Deadhesion

Formation and remodeling of disulfide bonds has been well studied for intracellular proteins in the cytoplasma, a compartment with a comparatively low redox potential due to redox-buffering cysteine and glutathione with a surplus of their reducing forms, cysteine and glutathione (GSH), as compared to their oxidized counterparts, cystine and oxidized, homodimerized glutathione (GSSG). This reducing redox environment is maintained by a high concentration of NADPH∙H^+^, which constantly reduces thioredoxin or glutaredoxin via thioredoxin reductase and glutaredoxin reductase. However, extracellular proteins are synthesized into the endoplasmic reticulum (ER), further processed and modified in the Golgi compartments, and transported and secreted in transport vesicles into the extracellular space or, in the case of membrane proteins, to the extracellular face of the cell membrane. Already at their first stage of production within the ER, they are exposed to an oxidative environment with a higher redox potential, which is necessary for a proper formation of disulfide bridges in a process called oxidative protein folding. This would require a high amount of cystine, GSSG or NADP^+^. However, the concentrations of such oxidants are scarce in the ER and oxidative protein folding relies on the oxidative power of molecular oxygen and its derivative hydrogen peroxide (H_2_O_2_). The predominant enzyme for the electron withdrawal from two cysteine residues in proteins is the endoplasmic reticulum oxidoreductin 1 (Ero1), which comes in two isoforms, Ero 1α and Ero 1β. It transfers the electrons via a flavin adenine dinucleotide (FAD) to molecular oxygen, thereby forming H_2_O_2_ [93]. A similar reaction is performed by the enzyme quiescin sulfhydryl oxidase (QSOX), which also comes in two isoforms, QSOX1 and QSOX2 [27]. Hydrogen peroxide, produced by either Ero1 or QSOX, has even greater oxidizing power and can be used by peroxiredoxin-4 (Prx4) to assist oxidative folding of secretory proteins by forming a disulfide bond via a temporary cysteine sulfenic acid, and water [27]. Interestingly, Prx4 is the only member of the six-member family of peroxiredoxins, whose genes encode a signal sequence directing its secretory translation into the ER.

**Figure 4:**
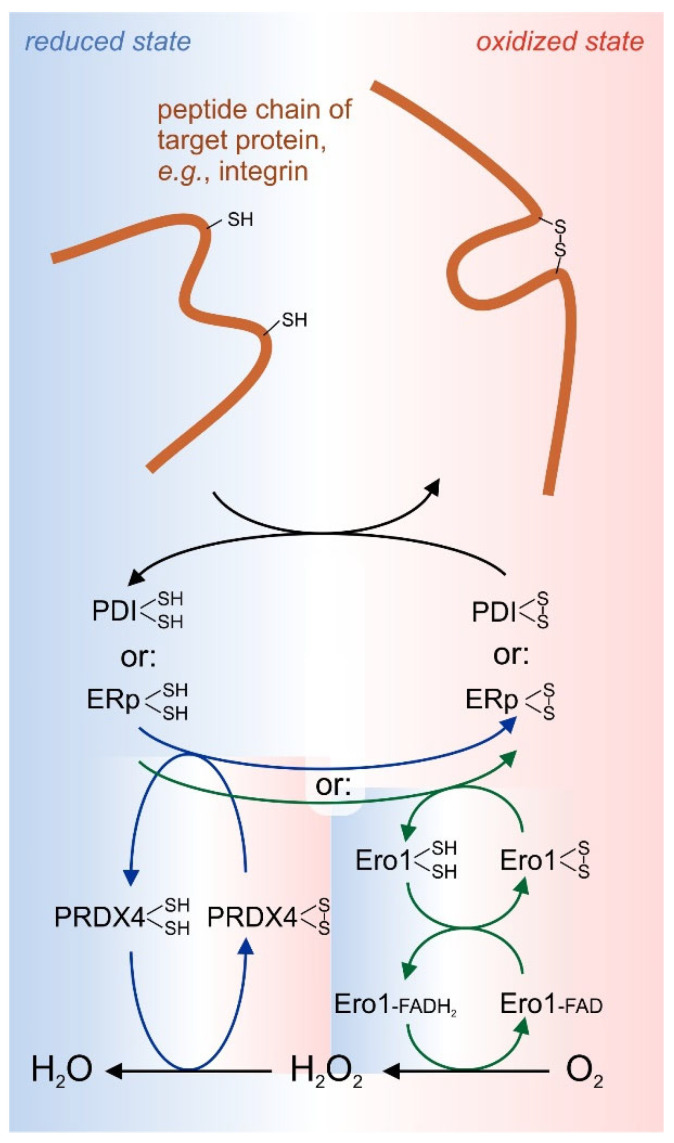
Redox chain for oxidative folding of proteins and the formation of disulfide bonds in secreted target proteins. This pathway occurs during the secretory pathway of extracellular proteins or of ectodomains of membrane proteins, such as integrin ectodomains. However, as several of these redox-modifying enzymes are also secreted into the pericellular space, it is likely that this oxidative redox chain to form disulfide bridges also takes place outside of cells. Alternative pathways starting with either molecular oxygen (via Ero1) or hydrogen peroxide (via peroxiredoxin-4, PRDX4) are shown in the bottom part. The Ero1-based redox circle can hypothetically be substituted for by the redox enzyme QSOX1, which is a membrane-anchored redox enzyme that is secreted onto the cell surface.

The oxidoreductases, Ero1 and QSOX, do not introduce disulfide bonds directly into the final target protein, but mainly use protein disulfide isomerases (PDI) as intermediate disulfide carriers that eventually oxidize the vicinal cysteine residues into a cystine bridge, while being reduced into dithiol forms that are recycled and reoxidized by Ero1 and QSOX [28] (Figure 4). A function similar to the one of PDI is fulfilled by so-called endoplasmic reticulum proteins (ERps), such as ERp5, ERp46, ERp72 and ERp57, the latter also known for its important role in the calreticulin-calcineurin pathway of ER stress and apoptosis [94]. PDIs and ERps form a large family of thiol isomerases with about 20 members. Their common feature is a thioredoxin-homologous domain (referred to as a or a’ domain) with the typical CGHC motif, which is usually present in tandem within the thiol isomerase, either connected next to each other (e.g., within ERp5, ERp72, and ERp46, the latter even in a triplet repeat) or flanking two connecting domains (commonly called b or b’ domain) that form the substrate protein binding site (e.g., in PDI, and ERp57). With an oxidized disulfide bridge, they approach the two spatially close cysteine residues of the substrate protein. These two vicinal cysteines are oxidized creating a disulfide bridge, while the CGHC motif of the thiol isomerase is reduced into two free thiol groups at the end of the reaction.

Most of the thiol isomerases and their redox-modifying partners carry the ER retention peptide sequence KDEL. However, in recent years, it has become increasingly clear, that at least six thiol isomerases are secreted into or escape into the peri- and extracellular space [26,28]. It is not yet clear how they can perform their oxidative function of forming disulfide bridges, as they are cut off from a constant supply of oxidants outside of the ER, such as those provided e.g., by Ero1. However, the extracellular form of QSOX1 may substitute for Ero1 in the peri- and extracellular environment [27]. The importance of extracellular thiol isomerases, which are stored in vesicles by platelets and endothelial cells and secreted in case of vessel damage, nowadays summarized as vascular thiol isomerases, has become more and more evident in the last few years. They play essential roles in platelet activation and blood coagulation. Moreover, they have even been identified as pharmacological targets in thrombosis [30,31,95,96].

So far, six vascular thiol isomerases have been identified that are secreted by platelets and endothelial cells into the extra- and pericellular space upon vessel damage [28,97,98]. They are PDI, ERp57, ERp72, ERp5, ERp46, and TMX1. The integrins αIIbβ3 and αVβ3 on platelets and endothelial cells, respectively, which belong to their target molecules, play a pivotal role in thrombus formation and stabilization. Several studies have highlighted the role of the secreted vascular thiol isomerases in coagulation and thrombus formation (reviewed in [3,28,30,31,99,100]). However, only a few studies showed a direct interaction of a particular thiol isomerase with the platelet integrin or integrin αVβ3 at the protein-chemical level [25,30].

PDI has a thiol isomerase activity on the platelet integrin [101]. This prototypic thiol isomerase, encoded by the gene prolyl-4-hydroxylase-β (P4HB), consists of two thioredoxin-like domains, a and a’, with a typical CGHC motif. They both flank two homologous domains, b and b’, the latter of which binds the substrate protein. A linker sequence between the b’ and a’ domains provides flexibility to the active site. Affecting platelet aggregation and adhesion to fibrin, PDI itself was identified to be redox regulated. H_2_O_2_ and endothelial cell-derived NO cause sulfenylation and nitrosylation, respectively, of thiol groups within PDI, thereby reducing fibrin binding of platelet integrin [95,102].

ERp57 (PDIA3) has a structure similar to PDI. It is an essential component of the oxidative folding machinery of the ER, and connects protein folding with the calreticulin-calcineurin pathway [103]. Although its role of disulfide formation in platelet integrin has not yet been precisely defined, the interaction of integrins with calreticulin in the ER makes its close association with ERp57 very likely. However, the extracellular role of ERp57 on platelet integrin regulation at the molecular level has remained elusive, although deficiency of ERp57 activity on platelets drastically reduces thrombus formation [104].

In addition to the basic a-b-b’-a’ domain structure, ERp72 (PDIA4) has an additional thioredoxin domain with a CGHC motif, named a°, that precedes the a-domain at its N-terminus. As an extracellular thiol isomerase, ERp72 oxidizes the long-range disulfide bridge (C654–C711, the numbering of the αM subunit, homologous to C490–C545 of the αIIB subunit) within the thigh domain of the αM subunit of the integrin αMβ2 (Mac1) on neutrophils. This interaction promotes integrin activation and neutrophil attachment to its ligand, the intercellular adhesion molecule-1 (ICAM1) on endothelial cells [105]. The homologous cysteine bond, C490–C545, within the αIIB subunit, is also redox-regulated and mostly not engaged in a disulfide bond but reduced with free thiol groups [106]. However, the corresponding thiol isomerase for the platelet integrin has not yet been identified.

ERp5 (PDIA6) has an extra a° domain in common with ERp72 but lacks the b’ and a’ domains. A platelet-specific ERp5 knockout shows increased secretion of other thiol isomerases in platelets and increased platelet adhesion to collagen, presumably via integrin α2β1 [107]. This suggests an attenuating effect of ERp5 on integrins. In fact, at the molecular level, ERp5 reductively cleaves the disulfide bridge C177–C184 within the A-domain of the platelet integrin β3 subunit causing its release from its fibrin ligand [108]. Interestingly, this occurs preferentially, after the platelet integrin has bound fibrin and transmits forces, which likely induce a conformational change within the integrin, thereby making it more accessible to ERp5.

In contrast, ERp46 (TXNDC5) activates the platelet integrin by cleaving the cysteine bridge C473–C503 within the hinge region of the integrin β3 subunit [109]. Worthwhile, ERp46 lacks any conventional substrate-binding b-domain and basically consists only of 3 thioredoxin-like domains with CGHC motifs [28].

The sixth member of the secreted thiol isomerases, the thioredoxin-related transmembrane protein-1 (TMX1) stands apart from the others, with respect to both structure and function [110]. TMX1 is a type I transmembrane protein, which contains one thioredoxin-like a-domain with a less homologous CPAC motif in its extracellular domain [31]. Because of its membrane anchorage, its action is limited to the pericellular space. Nevertheless, TMX1 is able to oxidize vicinal thiol groups within the platelet integrins, thereby inhibiting integrin-mediated platelet adhesion and migration [111]. As a negative regulator of integrin action, it counteracts the action of the other secreted thiol isomerases [31].

## 6. The Allosteric Disulfide Bridges of Integrins and Their Location

Which of the numerous disulfide bridges within integrins are redox-modified by the extracellular thiol isomerases? For the integrin αIIbβ3, they are listed in a recent review by Pijning et al. [25], including those that are of structural relevance. The latter are in the majority and are required for the correct folding and function. In fact, their mutations prevent the expression or function of a properly working integrin αIIbβ3. Thus, it may not be expressed or may have too low or high activity, resulting in bleeding disorders and constitutive activation with thrombosis, respectively, generally known as Glanzman thrombasthenia [25]. In most of these cases, cysteine residues within the EGF-domains of the β3 subunits are affected. Also, the four cysteines within the calf 2 domain of the integrin α7 subunit serve a structural role, among them the cysteine pair that crosslinks the heavy and light chain of the proteolytically cleaved integrin α subunit, as their replacement for alanine drastically reduced or abolished its expression [12,112]

The first evidence of allosteric disulfide bridges within the platelet integrin that serve regulatory functions redox dependently were reported 25 years ago [113]. Later, using the oxidizing “zero spacer” crosslinker phenylarsenic acid (PAO), it was demonstrated that a pair of such spatially close cysteine thiol groups does not necessarily form a disulfide bridge in the native protein, and it was shown that its PAO-induced formation has functional consequences [114]. Since then, potential thiol switches have been mapped at different sites within both subunits of integrin heterodimers. They are listed in Table 1.

The PSI domain contains a tandem array of two CXXC motifs, C_13_QQC_16_ and C_23_AWC_26_, which are similarly found in members of the PDI family (Figure 2B). The hypothesis that this integrin might not only be a substrate for redox modifying enzymes, but also might have PDI functions itself, was positively tested in protein–chemical assays [115,116]. In addition, antibodies directed against the cysteine-containing region of the PSI domain blocked fibrin binding to integrin αIIbβ3 and reduced platelet aggregation [116]. However, the PSI domain has only a low sequence homology to the typical CGHC-sequence of the thioredoxin and it does not resemble the thioredoxin-fold [117]. Moreover, in the crystal structure of the integrin αIIbβ3 in its bent conformation, the two cysteines in both CXXC motifs are neither reduced nor linked to each other. Interestingly, the first cysteine, C13 of the first CXXC motif, forms a long-range interdomain disulfide bond to cysteine C435 of the first EFG domain [25,74].

The A-domain of the β3 domain bears two cysteine pairs, C177–C184 and C232–C273 (Figure 2C). The former acts like a thiol switch, which is subjected to mechanical stress through ligand binding. Moreover, due to its surface exposure it is more easily reduced by the extracellular thiol isomerase ERp5 [108]. This finding is extremely interesting, as it links mechanical force transmission via integrin–ligand interaction to the accessibility and susceptibility of the cysteine bridge that leads to disengagement of two cysteine residues and enforced detachment of the platelet from fibrin [108]. Also within the integrin β2 subunit, the two homologous disulfide bonds C169–C176 and C224–C264, of the A domain of β2 in Mac-1, were identified to be allosteric disulfide bridges [118]. The first one is homologous to C177–C184 within the integrin β3 subunit. Reduction of the disulfide bonds within the β2 A-domain converts Mac1 (integrin αMβ2) into a less active conformation and consequentially to disengagement of Mac1 from its ligand ICAM-1, especially under shear forces of the blood stream. As PDI associates with Mac-1 at the trailing edge of neutrophils, PDI activity increases motility of neutrophils on the endothelial cell layer of capillaries and thus promotes extravasation of neutrophils [118].

Most disulfide bridges are located in the 4 EGF-repeats forming the leg of the integrin β subunit (Figure 2D). Two of the numerous disulfide bridges are of allosteric nature, C13–C435 and C437–C457. The first one connects the PSI domain (C13) with the N-terminal face of the EGF1 domain, while the second allosteric disulfide bridge, C473–C503, connects the C-terminal end of the EGF1 domain to the N-terminal end of the EGF2 domain, spanning the hinge region of the β3 subunit between the domains EGF1 and EGF2. Although not entirely assigned a redox regulatory role, the first intradomain disulfide bond of the EGF1 domain, C437–C457, is in close vicinity to the interdomain cysteine bridge between the PSI and EGF1-domain. The EGF1 and EGF2 domains flank the hinge sequence, around which the integrin head domain, together with the EGF1-domain, can pivot against the lower leg, the latter consisting of the EGF2 through EGF4-domains. Right within this hinge region, there is the disulfide bond C473–C503, which is highly surface exposed (Figure 2D) and is in a mechanically strained conformation [25]. It is reductively cleaved by the platelet thiol isomerase ERp46 [109]. Blocking of ERp46 with antibodies or knockout of ERp46 reduces binding of integrin αIIbβ3 to fibrin, platelet activation and thrombus formation.

**Table 1:** Potential allosteric disulfide bonds in integrin αIIBβ3, and homologous ones in other integrins.

Disulfide Bond	Domain Localization	Homologous Sites in Other Integrins	αC–αC Distance [nm]	Disulfide Strain Energy [kJ/mol] ^a^	Solvent Accessibility [Å^2^] ^a^	Stereochemical Conformation	Redox-Modifying Enzyme (and Effect)	References
C490–C545	αIIB thigh	C654–C711 in αM	0.42	19.2	6.59	-RH staple	ERp72 on integrin αMβ2 on neutrophils (promoting adhesion)	[25,119]
C602–C608	αIIB hinge	C589–C594 in α4 C606–C611 in murine α7 (X2 splice variant)	0.41	9.1	2.2	-LH hook	Reductive cleavage in α4 and disulfide bond formation in α7 promote ligand binding	[112,120,121]
C177–C184	β3 A	C169–C176 in β2 A	0.56	17.7	0.17	-/+RH hook	ERp5 (attenuating; reduction of disulfide bond under tension)	[108]
C232–C273	β3 A	C224–C264 in β2 A	0.52	10.0	8.56	-RH hook	PDI (attenuating binding affinity)	[118]
C13–C435	Interdomain β3 PSI-β3 EGF1		0.66	15.7	1.70	+/-LH spiral	unknown	
C473–C503	Hinge between EGF1 and EGF2		0.68	48.4	27.37	-/+RH hook	ERp46 (reductive cleavage, activating integrin)	[109]
C437–C457	EGF1 of β3	C494–C526 in β7	0.54	17.2	19.90	-/+RH hook	in αVβ3 and in α4β7 (in the latter, reductive cleavage activates integrin)	[88,90,121,122]
C523–C544	EGF3 of β3 (at interface with EGF2)		0.41	17.3	3.36	-LH hook	Putatively ERp57 (inhibiting αIIbβ3, but not αVβ3)	[88,90,122]

^a^, taken from [25].

Also highly surface-exposed and under mechanical strain is the disulfide bond C523–C544, the first disulfide bridge within EGF3, which is located at the interdomain face towards EGF2 within αIIbβ3 [25]. Replacement of this pair of cysteines with redox inactive serine residues resulted in a constitutively active platelet integrin, whereas the same mutation was ineffective within the integrin αVβ3, albeit containing the same β subunit [90,123]. A systematic replacement of additional cysteine residues within the EGF domains was carried out, but as the outcomes depended also on the combination with other mutated cysteine residues, this has not yet provided a clear picture of which cysteines, apart from the above named ones, are of structural importance or have an activity-regulating, allosteric effect on integrins. The thiol isomerase ERp57 has been hypothesized to be active at these EGF sites [88,90,122].

The integrin α subunits possess less cysteine residues than the integrin β subunits. However, exposure of the recombinant α7β1 integrin ectodomain to hydrogen peroxide, a typical member of the reactive oxygen species (ROS), revealed thiol oxidation products of cysteines, other than disulfide bridges, within the integrin α7 subunit only, but not the β1 subunit. They were mapped to the hinge and calf 2 domain [12]. Similarly, differential cysteine alkylation studies on the platelet integrin revealed that the long-range cysteine bond C490–C545, within the thigh domain of the αIIB subunit, is not formed in one of three cases, but remains in the reduced form [13,25]. It spans two β strands of the thigh domain and thus appears to be of structural importance. Nevertheless, mutation of these two cysteines into non-crosslinkable alanine residues resulted in less cell spreading and enhanced recruitment of the mutated integrin into adhesion complexes [106,119]. As these supramolecular adhesive structures are located preferentially underneath the nuclei, not in the cell periphery, they appear to be fibrillar adhesions that mediate firm and static cell adhesion rather than focal adhesion plaques of lamellipodia that are involved in cell migration [38,55,56]. Interestingly, such behavior of this mutated integrin was not caused by an altered reaction of the ectodomains but by the reduced association with adaptor protein-2 (AP2) towards the cytoplasmic domain, which is relevant for clathrin-dependent integrin recycling. Its mutation-induced reduction of AP2 association failed to renew focal adhesions in the periphery of cells, along with reduced lamellipodia formation and decreased spreading on an adhesive substratum [106,119]. As a consequence, abundant surface integrins were recruited into fibrillar adhesions. Likewise, the homologous cysteine bond, Cys654–Cys711, within the thigh domain of the αM subunit of integrin αMβ2 (Mac1) is cleaved by ERp72, thereby promoting neutrophil granulocytes to adhere to their cognate ligand on the endothelial cells [105].

Although not experimentally addressed in the platelet integrin αIIbβ3, the cysteine pair within the hinge region between the thigh and calf 1 domain, which is highly conserved throughout all integrin α subunits, forms an important allosteric disulfide bond. This was proven for the α4 and α7 subunits of the integrins, α4β7 and α7β1, respectively [112,121]. The cysteine bridge of the integrin α7 subunit serves as a thiol switch, as its formation by hydrogen peroxide increases its affinity towards its ligand laminin-111, induces a structural conversion from its bent to the more active elongated conformation, and promotes migration of integrin α7β1-bearing cells on laminin-111 [112]. The oxidant H_2_O_2_ is formed at the adhesion sites of cells by NADPH-oxidase 4 (NOX4) and thus activates the integrin for cell adhesion [12]. The chemical crosslinking of the two free thiol groups of the cysteine pair with a homobifunctional crosslinker resulted in a similar integrin activation and provided evidence that the formation of the disulfide bridge within the hinge region of the α subunit initiated the formation of a chelation site for a divalent cation, most likely Ca^2+^, which is essential for ligand binding [120]. The paramount importance of this thiol switch was also shown for the integrin α4 subunit in a double mutant by concomitantly mutating the cysteine bridge in the β7 subunit spanning the hinge region between EGF1-and EGF2 [121]. The latter is also conserved within the different integrin β subunits and is homologous to the cysteine bond C437–C457 within the β3 subunit of the platelet integrin. However, in contrast to our work [112,120], Zhang et al. (2013) showed that a reductant, dithiothreitol (DTT), rather than the oxidizing H_2_O_2_, increased ligand binding of α4β7 integrin [121]. Conspicuously, each of the hinge regions of both integrin subunits α and β, between the thigh and calf 1 and between the EGF1 with its adjacent PSI and EGF2 domains, contain a redox-sensitive cysteine pair. As the hinge region is spatially distant from the ligand-binding head domain of integrins, these two thiol switches are of clear allosteric nature. Their influence on ligand binding and integrin activation must be conveyed via conformational changes.

## 7. The Formation and Cleavage of Allosteric Disulfide Bonds in Integrins Causes Conformational Changes

By forming a disulfide bridge, the distance between the αC-atoms, and thus of the protein backbone chain, is fixed in a distance range of 0.43 to 0.65 nm [16]. How can this covalent fixation across such a short distance lead to a signal? A conceivable way to amplify this subtle structural change of a sub-nanometer scaled bond is the use of a lever structure within the protein. Indeed, such amplification is achieved by mechanical movements within the protein, such as piston-like shifts of secondary structure elements, *viz.* α-helices, against each other in combination with rotational movements of tertiary structural elements and domains around hinges within the integrin. Molecular structure analyses have revealed such a piston shift of two α-helices in the A domain of the integrin α subunit [75,76]. Moreover, a rotational movement, in which the head piece together with the upper legs pivots against the lower leg domains, amplifies the distances. The pivot is the integrin hinge region, also known as the knee region, which is flanked by the thigh and calf1 domains, and by the EGF1 and EGF2 domains within the integrin α and β subunits, respectively [74,124] (Figure 1). Conspicuously, the redox-regulated disulfide bridges of integrins are located in these conformationally relevant domains, the A-domain of the α subunit and the hinge regions of both α and β subunit, including the domains adjacent to the hinge regions. The hinge regions are not part of the ECM ligand binding site, which is mapped to the A-domain of the integrin α subunit and, in case of αA-domain-lacking integrins, to the α subunit propeller and the β subunit A-domain [56,125].

**Figure 5:**
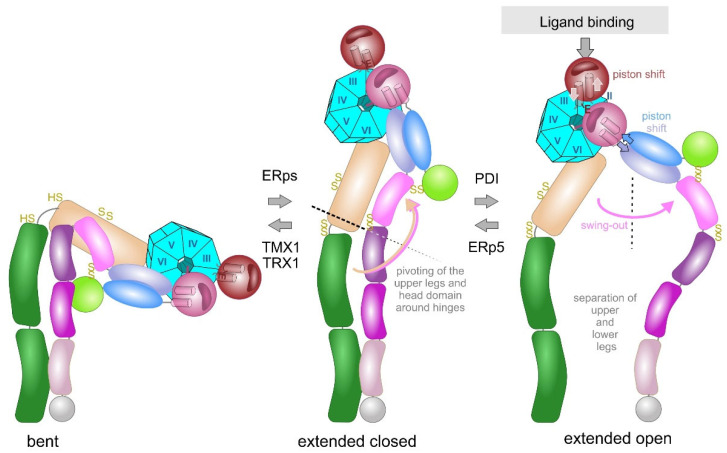
Conformational changes within the integrin ectodomain structure and the potential involvement of allosteric disulfide bonds. A two-step model of conformational changes of an integrin is shown. The first step is the extension of the ectodomain from a bent to an extended closed conformation by jointly rotating the head piece and upper leg regions around the hinges away from the lower legs. The second molecular movement is the swing-out movement and separation of the upper legs of both subunits. This eventually separates the transmembrane and cytoplasmic domains. These conformational changes convey the signal of an ECM ligand binding to the cytoplasm during outside-in signaling. The involvement of redox-modifying enzymes in the conformational changes are indicated. TRX1, thioredoxin1.

The rotational movement around the hinge region is an essential part of the extension model, in which the integrin changes between a bent and extended conformation (Figure 5). In a reversible manner, the bent conformation, in which the ligand binding head domain points towards the cell membrane, converts into the extended conformation, in which the entire integrin ectodomain takes an upright shape with the ligand binding site pointing away from the cell membrane towards the ECM [124,125,126,127]. Electron microscopy, combined with single particle analysis, and small angle x-ray scattering proved the extension of the integrin upon activation in vitro [124,128,129] and on the cell surface [130]. Additionally, conformation-dependent antibodies helped to define specific integrin conformations [131]. During extension, contacts between the upper and lower leg domains, as well as between the head piece and the lower leg domains are loosened, especially the intra-β-chain interactions between the βA and βTD domains [80] and the inter-subunit contacts between the membrane-proximal calf 2 and EGF4 domains [132].

During the extension of the integrin, the hinge regions of both subunits still remain together, so that the bent conformation converts into the so-called extended close conformation with an increased binding affinity to its ECM ligand. Subsequently, ligand binding triggers another conformational change of the integrin ectodomain, in which the knee/hinge regions of both integrin subunits separate. This so-called swing-out movement converts the extended closed conformation into the extended open one with the latter having the highest affinity to its ECM ligand [82,133,134]. Interestingly, the conformations differ thermodynamically, as the free enthalpy rises strongly upon integrin extension and slightly from the extended closed to the extended open conformation rendering integrin activation an energy-demanding process [135].

**Figure 6:**
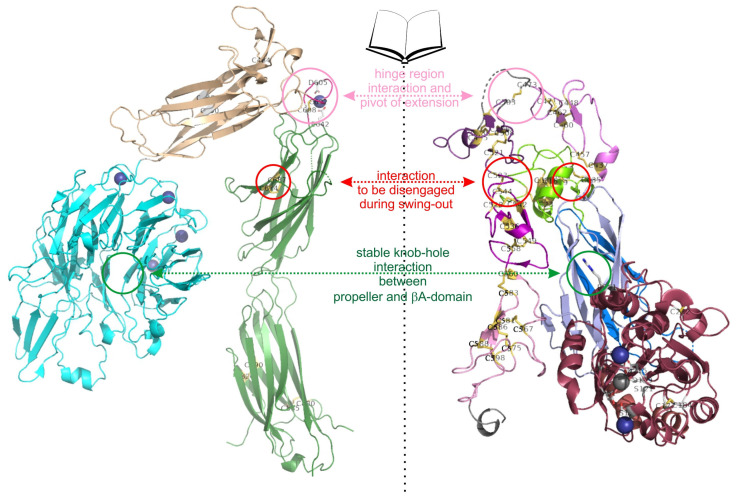
Interfaces between the two integrin subunits in a bookview presentation. The stable interaction between the two subunits is achieved by the protrusion of a basic amino acid side chain (R261 in β3) that serves as a knob pointing into the central pore of the propeller domain (encircled in green). This tight interaction provides a structurally robust platform of the integrin head piece. Other interactions between the two subunits occur in the hinges, which are the essential pivots for the extension movement of the ectodomain. During the swing-out movement, the interactions between the calf 1 and EGF2/3 and hybrid/PSI domain of the upper legs are loosened. Conspicuously, allosteric disulfide bonds are mapped to these interfaces within the hinges (C602–C608 in the α subunit hinge and C473–C503 of EGF1 and EGF2 in the ß subunit) and the upper leg regions (C490–C545 in the thigh domain and disulfide bonds in PSI, EGF1, and in EGF3) (encircled in red). The molecular structure was rendered with Pymol Molecular Graphics System software, version 2.3.1, using the protein data file 3FCS.pdb of RCSB Protein Data Bank.

Ligand binding triggers the second conformational change, the swing-out movement of the upper leg regions (Figure 5). In the αA-containing integrins, the ligand, such as collagen, binds via the MIDAS to the top of the αA-domain. This binding crevice is opposite to the N- and C-terminal linker sequences of the αA-domain, by which it is connected to the propeller domain. The major structural consequences are unfolding of an additional α-helix C, a shift of the ADMIDAS metal ion close to the binding crevice and a piston movement of α-helix 7 against α-helix 1 by about 1 nm [56,76,133]. As a consequence of the ligand-induced piston shift of the α-helix 7, a glutamate residue in its C-terminal adjacent linker-region approaches the MIDAS of the neighboring βA-domain and serves as an “internal” ligand [136]. During this process, the residue R261 on the side face of the βA domain holds it in place towards the propeller domain, by poking its side chain deeply into the central pore with the propeller domain (Figure 6). This interaction likely contributes to the stable platform of the head piece, consisting of the βA-domain and the propeller domain as well as the αA-domain, if present. From the head piece, both flexible legs protrude. Whereas in αA-domain-containing integrins the glutamate residue that stands out from the αA domain serves as an internal ligand for the βA-domain, this role is taken by an acidic amino acid side chain of an ECM ligand in αA-domain-lacking integrins. For example, this can be the aspartate side chain of the RGD-motif in fibrin [77,137]. Then, this likely leads to a piston shift between α-helices 1 and 7, similar to the one within the A domain of the integrin α subunit [138].

How could the cysteines within the A-domains affect the conformational changes? In the A-domain of the integrin α subunit, the two cysteines are too far apart from each other, separated by the central β-sheet and hardly accessible from the protein surface to be oxidized into a disulfide bridge. In contrast, the two disulfide bridges within the βA domain, C177–C184 and C232–C273, are redox-regulated and located at conformationally relevant sites. Whereas C177–C184 is located at the top of the βA domain, close to the ligand-binding crevice, but not directly participating in the loops that shape the metal-binding sites (MIDAS and ADMIDAS), the second disulfide bridge, C232–C273 of the βA domain is situated in a loop structure between α-helix 4 and β-strand D, which also harbors the R261 residue that anchors the βA domain to the propeller domain [70,77]. It can be envisioned that opening the disulfide bridge C232–C273 results in a loosening of this static interdomain platform of the βA-domain with the propeller domain, thereby affecting the mechanics of secondary structure movements.

Since both A-domains are directly connected to the propeller domain within the head piece, displacements of the pistons within these domains causes the piston shifts to be conveyed via the rod-like hybrid domain towards the hinge domain. As the βA domain is inserted into the hybrid domain, there are two peptide strands connecting the two domains. Along the N→C direction, the peptide chain enters helix 1 of the βA domain from the hybrid domain, while it leads out via helix 7 of the βA domain back into the hybrid domain. The piston displacement of helices 1 and 7 via these two connections is thereby transmitted into a rotational movement of the hybrid domain, which causes it to swing out like a lever, which is further amplified by the adjacent EGF1-domain [139].

The EGF1 domain contains two allosteric disulfide bridges, which are located at either end of the domain. The first one, C13–C435, connects the PSI domain with the EGF1, while the latter, C475–C503, links EGF1 to EGF2 in the hinge region [134,139,140]. Therefore, it can be hypothesized that both disulfide bridges have a significant impact on the swing-out movement of the integrin upper legs from the head piece.

The hinge domains of both subunits play a role not only in the extension but also in the swing-out movement, which eventually separates the knee regions of both integrin subunits. The thiol switch within the α subunit hinge region is of dominant importance in this conformational conversion [112,120,127]. The swing-out movement goes along with the disengagement of the interfaces between the two integrin subunits. In the bent and extended-closed conformation, before the swing-out, the upper legs of both subunits still remain close together with several molecular interactions [73], as the thigh domain interacts with the hybrid and EGF1 domain. Also, the calf 1 and 2 domains form interfaces with the EGF-domains 3 and 4 (Figure 6). It is noteworthy that the disulfide bridge C523–C544 of EGF3 domain of the β3 subunit comes into the vicinity of the disulfide bridge C674–C687 of the calf 1 domain of the αIIb subunit. Likewise, the thigh domain and the hinge domain of the integrin α subunit also contain the redox-regulated disulfide bridges C490–C545 and C602–C608, respectively. They are close to the EGF2 domain of the β-subunit, which loses its contact with the α-subunit thigh domain upon separation of the upper legs [73,133,140].

As a consequence of the swing-out movement, the separated stalks push the two transmembrane and cytoplasmic domains of both integrin subunits apart. This conformational change conveys signals and converts them into the association of adaptor and signaling molecules to the cytoplasmic tails. Of pivotal importance, the conformational conversion correlates with the biological functions of the integrin that acts as anchoring receptor and signaling relay within the cell membrane [44,66,133].

## 8. From Disulfide Bridge-Induced Conformational Changes to Cellular Consequences of Integrin-ECM Contacts

The oxidative formation and reductive cleavage of disulfide bridges within integrins cause conformational changes. This alters their affinity towards the respective ECM ligands. In addition, ECM occupancy of integrins induces clustering of integrins [51], or if integrin clusters already exist before ligand binding, eventually induces recruitment of adaptor and signaling molecules to the separated integrin cytoplasmic tails. Thus, a new adhesive cell organelle termed an adhesome is formed [58,62,141]. In its highly ordered layer structure, cytoskeletal adaptor molecules and regulator proteins interacting with the cytoplasmic integrin tails form the connection from the actin fiber network and its motor proteins to the ECM [142]. Thus, adhesomes serve as both mechanical anchorage points and signaling hubs.

However, it must be stated that redox-active compounds and redox-modifying enzymes are not the only factors that affect integrin binding to their ECM ligands or influence integrin association with intracellular adaptor and signaling molecules. Also, other parameters, such as concentration of divalent cations, tensile forces and intracellular adaptor proteins, are also valid effectors of integrin activity. Divalent metal ions may serve both structural and regulatory functions within integrins [78,143]. Some of the divalent cations, such as the ones of the bottom face of the propeller domain likely keep up a structure, whereas the divalent cations within the A-domains are essential to bridge the ECM with the integrin during binding [78,143]. Within the α subunit hinge region, the divalent cation binding site depends on the formation of the disulfide bridge [120]. Tensile forces that are transmitted via the integrins across the cell membrane also influence integrin activity [56,144,145]. In addition, the rate of force exertion is important, as integrins form a catch bond to their extracellular ligand. It also has an effect on integrin conformation [146,147]. Moreover, the structure and composition of an adhesome changes with the load and duration of the integrin-mediated forces [56,148]. Also regulating integrin activity, intracellular adaptor proteins, such as kindlin and talin, activate integrins during inside-out signaling and thus trigger cell attachment [149,150]. Upon binding to the cytoplasmic tail of the integrin β-subunit, kindlin and talin keep integrins in the extended open conformation with high ECM ligand-binding activity [37,149]. Their association with integrins depends on small G-proteins, the corresponding guanine nucleotide exchange factors (GEFs), and GTPase-activating proteins (GAPs) [150,151,152,153].

Besides the fact that several different factors influence integrins, it must also be stated that integrins are not the only components of the supramolecular adhesome complex that are redox regulated [59,60]. Therefore, redox regulation of integrins via their thiol switches has to be seen as part of a complex regulatory and signaling network that orchestrates cell adhesion, migration and other integrin-related cellular functions, such as anchorage-dependent growth and survival, as well as differentiation [25,154,155].

Being anucleate cell fragments, platelets and their hemostatic function to stop blood leakage from injured blood vessels have been more intensively studied for redox regulation of integrin-mediated functions. A common target of vascular thiol isomerases is integrin αIIbβ3, the fibrin (ogen) receptor, which, with 80,000 copies per platelet, is by far the most abundant integrin receptor [35]. Among the numerous stimuli of platelet activation, the contact of platelets with immobilized ECM components, such as collagen, indicative of vessel damage, activates integrin α2β1, the sole collagen-binding integrin on platelets, in a redox-dependent manner [156]. Via inside-out signaling, activated platelets convert platelet integrin αIIbβ3 into a highly active receptor for fibrin. Fibrin is the end product of the coagulation cascade, and its network stabilizes the newly formed thrombus. Both platelet integrin and fibrin are redox-regulated molecules, along with several factors of the coagulation cascade, such as the Tissue Factor [21,49,157]. Particular disulfide bridges within these molecules are the molecular redox switches, which are targets for cognate vascular thiol isomerases [30,96,97].

Redox regulation is not only restricted to the anucleate platelets but also occurs in numerous cells with different integrins. In addition to αIIbβ3, disulfide-based redox regulation has also been described for the collagen-binding integrins, α2β1 and α11β1 [156,158], for the laminin-binding integrin α7β1 [12,112,120], and for the immunologically relevant integrins, α4β1, α4β7, αMβ2, and αLβ2, on leukocytes [118,121,159,160,161,162,163]. In most of these cases, PDI or other thiol isomerases are involved, and even physical association of them with the respective integrin was proven [30,104,158]. They influence the ECM ligand-binding affinity positively [30,59,104,154,158] or negatively [108]. Mostly, the thiol isomerases use an oxidant, e.g., molecular oxygen or H_2_O_2_, to form a disulfide bridge. As an exception, the thiol isomerase TMX1 reduces disulfide bridges, but has so far only been seen to be operative on the platelet integrin [111]. Also, reduced thioredoxin-1 can reduce α7β1-mediated cell migration by cleaving its hinge thiol switch of its α7 subunit [112]. Albeit found in the extracellular space [3,164], thioredoxin-1, however, would need a constant supply of NADPH∙H^+^ in the pericellular space to be reduced by thioredoxin reductase and to maintain a redox regulation-supporting electron transport chain.

By altering integrin conformation, disulfide-based redox regulation of integrin enables the cells to regulate or fine-tune their capabilities to transmit forces and to migrate. In order to adhere and spread, cells have to exert forces onto their ECM substrate. The binding of integrins to their ligands withstands decent forces [165,166,167,168,169,170]. Moreover, maximum force load transmitted by integrins is regulated by the redox modification of their allosteric disulfide bridges [57,118,144,171]. Vice versa, mechanical force-mediated conformational changes of integrins may make particular disulfide bonds more accessible to thiol isomerases and thus influence their redox modification [108]. The mechanical forces transmitted via integrins may not only facilitate firm cellular adhesion, but may also be how cells set the ECM under tension, a biophysical parameter that influences the behavior of other cells in tissues via integrin-mediated mechanosensing [38,172].

Cell migration is a multi-step process, which includes integrin engagement with the ECM ligand and force transmission at the cell front, but also the detachment of the cell and retraction at its rear end as well as recycling of disengaged integrins from the rear to the front end of the cell [54,173,174,175]. A redox-mediated decrease of integrin-binding affinity was demonstrated for the integrin αMβ2 at the posterior end of neutrophil granulocytes, thereby promoting their migratory extravasation [118]. Also, redox modification of integrins within their thigh domain by thiol isomerases affects integrin recycling. Consequently, longer retainment of integrins changes their location from migration-supporting focal adhesions to fibrillary adhesions, a different adhesome type which favors firm cell adhesion [25,106].

## 9. Concluding Remarks and Future Perspectives

Although the platelet integrin and its involvement in thrombotic events has been the focus of studies on redox regulation of cell adhesion, all integrin-related cellular functions, including in nucleate cells, are redox regulated. We are only just beginning to understand the redox regulation of integrins and their adhesome structures in various (patho)physiological processes, such as blood coagulation, vascular remodeling, mechanosensing, endothelial function, immune responses, inflammation, tumor progression and metastasis [3,59,176].

What is the clinical relevance of the finding that integrin functions are redox regulated? How may this discovery lead to tangible applications in medicine? At this time, the answers to these questions may remain vague, but eventually will become clear. To substantiate the answers, we need to know more about the redox potential of cell compartments, tissues and whole organs. The local oxygen tension is an important factor, but also the special and temporal presence of oxidants, reductants or antioxidants, and redox-modifying enzymes will be parameters used to draw a clearer map of redox potentials within the body. Different diets with provision of antioxidants, e.g., ascorbic acid or vitamin E, and lifestyle choices, e.g., smoking, change the redox environment in tissues [177,178,179]. In fact, the redox potential of the blood of smokers is measurably higher than that of non-smokers [180]. Immunological diseases, such as pathogen defense and arteriosclerosis, are also accompanied with increased conversion of molecular oxygen, known as respiratory burst, and the production of ROS and RNS, altogether resulting in a locally increased redox potential, which effect immune cells at large [181] and integrin-related function of immune cells in particular, such as diapedesis, intrastromal migration of immune cells to the inflammation site, and phagocytic uptake of opsonized pathogens. Studies so far show that redox regulation of integrin exists and affects migration of leukocytes [105,108] and adhesion of platelets [30] at the molecular and cellular level. However, these studies have to be extended to the tissue and organism level, which then will provide a clearer picture of how redox regulation of integrins can be influenced by antioxidants and other dietary factors. Moreover, new therapeutics targeting the redox-modifying enzymes that act on integrins can be developed to reduce thrombosis and to manipulate inflammatory processes in the future.
